# A cross-sectional study on the response abilities of clinical and preventive medical students in public health emergency

**DOI:** 10.3389/fpubh.2022.1017063

**Published:** 2022-12-01

**Authors:** Yao Yu, Yixuan Qin, Yuxuan Liao, Zijiang Yang, Puqiao Wen, Jianzhen Wu, Pengfei Rong

**Affiliations:** ^1^Department of Health Management, The Third Xiangya Hospital, Central South University, Changsha, China; ^2^Xiangya School of Medicine, Central South University, Changsha, China; ^3^Xiangya School of Public Health, Central South University, Changsha, China; ^4^Department of Radiology, The Third Xiangya Hospital, Central South University, Changsha, China

**Keywords:** public health emergency, clinical medical students, preventive medical students, training program, education reform

## Abstract

Inconsistent training programs for public health emergency (PHE) have been criticized as a contributing factor in PHE's managerial weak points. In response, to analyze the relevant discrepancies among the medical students in the class of 2021 from Xiangya School of Medicine of Central South University, the present study conducted an online questionnaire survey using convenience sampling. The questionnaire comprised four sections, including the basic information, the subjective cognition in PHE, the rescue knowledge and capabilities of PHE, and the mastery of PHE regulations and psychological intervention abilities. To compare the abovementioned aspects, related data were collected from 235 medical students divided into two groups, namely, clinical medical students (Group A) and preventive medical students (Group B). We found a more positive attitude in PHE (*P* = 0.014) and a better grasp of the PHE classification (*P* = 0.027) and the reporting system in group B compared with group A. In addition, even if group B showed the same response capability in communicable diseases as group A, the former had less access to clinical practice, resulting in poorer performance in the noncommunicable diseases during a fire, flood, and traffic accidents (*P* = 0.002, *P* = 0.018, *P* = 0.002). The different emphasis of each training program contributed to the uneven distribution of abilities and cognition. Meanwhile, the lack of an integrated PHE curriculum led to unsystematic expertise. Hence, to optimize the PHE management system, equal attention should be paid to medical students with diverse majors along with a complete integrated PHE curriculum.

## Introduction

Public health emergency (PHE) refers to major infectious diseases, population unexplained diseases, major food and occupational poisonings, and other events that occur suddenly and may cause severe harm to public health (1, 2). It possesses the characteristics of explosivity, harmfulness, contingency, publicity, and enforceability. In the context of globalization, the incidence of PHE is increasing in China, along with the outbreaks of acute infectious diseases that have broadly adverse impacts on individuals as well as society, further threatening the national wellbeing, such as the severe acute respiratory syndrome (SARS), H1N1, H7N9, the Middle East respiratory syndrome (MERS), and the Coronavirus disease 2019 (COVID-19), which continues to disseminate globally (3–6). Consequently, actions must be taken to institute a sound scientific PHE management system.

In the fight against COVID-19, the Chinese government has become an indelible leading light with the forceful PHE management system (7–9), deploying comprehensive medical teams that have specialists in the fields of basic medical sciences, clinical medicine, and preventive medicine as before. Conversely, it exposed various problems, such as deficiencies in the complete cognitive systems and general coping capacities among professionals (9). The phenomenon fully reflected the fact that professionals with theoretical knowledge and practical capabilities remained a small part of the system, which posed a major challenge to the PHE management system (10). Hence, how to systematically cultivate and strengthen the knowledge and coping abilities of medical students responding to PHE has become a pivotal question of practical significance in medical education.

In Chinese undergraduate medical education, the training program for students of clinical medicine aims to cultivate distinguished doctors who are responsible for rescuing patients, while the program for students of preventive medicine intends to cultivate public health specialists who are responsible for stopping transmission (11). The professional core curriculum of clinical medicine mainly includes clinical courses such as surgery, internal medicine, uterology, pediatrics, and lemology, occupying an enormous percentage of total credits (12). Meanwhile, the curriculum of preventive medicine mainly incorporates preventive courses such as epidemiology, biostatistics, and occupational health and medicine. Moreover, preventive medicine students are required to not only master the main courses and basic medical sciences but also learn clinical medicine, most of which are compulsory courses with fewer credits (13). Fewer course credits in Chinese education always represent lower requirements compared with the higher ones, showing that their knowledge of these courses is comparatively superficial (14). By contrast, clinical medical students are not required to learn preventive medicine, most of which are elective courses with fewer credits (15). In other words, there is almost no PHE-related curriculum and no integrated curriculum for both students to master the PHE management system, which only exists in various courses separately (16). Hence, students with limited ability to integrate fragmentary knowledge together can hardly form a consistent cognition toward PHE and respond in an overall manner, leading to certain defects in the PHE management system. To sum up, the loss in PHE education and unilateral training program causes discrepancies in subjective cognition in PHE, relevant knowledge, and coping capabilities between the students of clinical medicine and those of preventive medicine, leading to further effects on the PHE management system.

To better understand the emergency cognition in PHE and the current state of related knowledge of medical students in different majors, potential problems in medical education were analyzed, and weak links and influencing factors were identified to cultivate students' emergency knowledge and practical capabilities. To further optimize the medical education system, an online questionnaire survey was conducted using convenience sampling among medical students in the class of 2021 from Xiangya School of Medicine of Central South University. Analyzing the data ensured the detailed impacts of the different training programs on the students' cognition and skills. In addition, we put forward several relevant suggestions based on the findings.

## Method

### Ethical approval

The Third Xiangya Hospital Ethics Association of Central South University approved the study, and all participants signed the informed consent forms as the ethics association required.

### Study design and specific questionnaire design

This was a cross-sectional study on the response abilities of clinical and preventive medicine students in PHE with the method of convenience sampling survey. The questionnaire comprised four sections, including the basic information, the subjective cognition toward PHE, the rescue knowledge and capabilities of PHE, and the mastery of PHE regulations and psychological intervention abilities.

The first block collected demographic data, including gender and major. It also covered some basic information, such as whether he/she has systematically gained theoretical knowledge of PHE, whether he/she has participated in the emergency exercise of PHE, and whether he/she has experienced rescue activity of PHE. The second block reflected their subjective cognition in PHE including 6 questions, such as their psychological states during PHE, their will to participate in PHE rescue, their views on the necessity of PHE courses and training, their cognition of the necessity of PHE exercise, and so on, using the 5-point Likert scale. Their attitudes were graded as follows: (1) totally disagree; (2) disagree; (3) uncertain; (4) agree; and (5) totally agree.

The third block estimated their knowledge and capabilities of PHE rescue. The interviewees were required to evaluate their knowledge and capabilities of noncommunicable diseases (including the prehension of trauma assessment scale, the knowledge of ambulance during fire disasters rescue, the knowledge of ambulance during flood disasters rescue, the knowledge of casualty assessment and rescue in serious traffic accidents, and the knowledge of identification and rescue of food poisoning) along with their knowledge of the communicable diseases (including fever clinics, COVID-19, H1N1, and respiratory communicable diseases.) using the 5-point Likert scale. Their knowledge and capabilities were graded as follows: (1) terrible; (2) poor; (3) average; (4) good; and (5) excellent.

The fourth block assessed their mastery of PHE regulations (classification management, reporting system, and laws) together with psychological intervention abilities using the 5-point Likert scale. Their capabilities were graded as follows: (1) terrible; (2) poor; (3) average; (4) good; and (5) excellent.

### Questionnaire distribution and recovery

All participants were 2021 graduates from Xiangya Medical School of Central South University, majoring in clinical medicine (*n* = 370) or preventive medicine (*n* = 86). In September 2021, the questionnaires were distributed to all participants online *via* the Wenjuanxing platform (https://www.wjx.cn/). We totally collected 250 questionnaires, 235 of which (189 from clinical medical students and 46 from preventive medical students) with reliable and valid answers were used for research. The ratio of clinical medicine students to preventive medicine students was four to one, which was consistent with the composition ratio of the questionnaire we collected. Therefore, we considered that the gathered questionnaires had high representativeness.

### Questionnaire reliability analysis

The content of the questionnaire was reviewed by an expert panel, including a medical education specialist, a student affairs management officer responsible for the clinical and preventive undergraduates' training programs, an associate professor of health statistics, and an associate professor of the epidemiological specialist. We also pretested the survey with 30 students and revised the questionnaire according to the feedback. Cronbach's α of the total scale in this research (0.941) was examined for testing the internal consistency reliability.

### Questionnaire data grouping

The purpose of this study was to make a statistical inference by analyzing the current difference in PHE knowledge between clinical and preventive medicine students to optimize the undergraduate education system. Accordingly, the subjects were divided into two groups. Students in group A graduated from clinical medicine, and those in group B graduated from preventive medicine. Clinical medicine students are required to master basic medical practice abilities, while preventive medicine students are required to master public health-related medical practice abilities. Both being medical students, they share the same courses such as internal medicine, uterology, pediatrics, lemology, epidemiology, and health law. Nevertheless, the grades, credits, credit hours, and requirements for those courses differ from each other. For instance, the grade of pediatrics in the clinical curriculum was A, while the grade in the preventive curriculum was B, and the credits were 8.5 and 4, respectively. Meanwhile, the grade of epidemiology in the clinical curriculum was B, while the grade in the preventive curriculum was A, and the credits were 2 and 4.5, respectively. In Chinese universities, 1 credit always represents 16 credit hours, and 1 credit hour is equivalent to 45 min. In addition, the courses with higher credits do a large proportion of the overall score, and the standard of examination is much more demanding, encouraging students to put more effort into those courses.

### Statistical analysis of questionnaire data

SPSS 26.0 was used for data analyses. We first used the Kolmogorov-Smirnov test to analyze the normality of the data, which showed that it was a skewed distribution. Therefore, non-parametric tests were used for comparing the difference between the two sets of data, including the subjective cognition toward PHE, the rescue knowledge and capabilities of PHE, the mastery of PHE regulations, and psychological intervention abilities. The chi-square test was used for comparing the constituent ratios of the demographic data and basic information between groups A and B. A *P*-value of < 0.05 was considered statistically significant.

## Results

### Demographic characteristics and basic information

For further analysis, 1 week after the questionnaires were distributed, 235 (94.0%) of the questionnaires with reliable and valid answers were used, including 189 (94.5%) clinical medicine students (Group A) and 46 (92.0%) preventive medicine students (Group B). In group A, 76 (40.2%) of them were men and 113 (59.8%) of them were women; meanwhile, group B consisted of 10 (21.7%) men and 36 (78.3%) women, which was consistent with the sex ratio between the two majors from XiangYa Medical School of Central South University.

The results showed that, compared with group A (82.0%, *P* = 0.021), group B (95.7%) learned PHE theoretical knowledge more systematically. However, group B had no experience of PHE rescues (0.0%, *P* = 0.002) while group A had 75.3%. Moreover, although not statistically significant, it was clear that group A students in fact had more opportunities to participate in PHE exercise (32.3%) than group B (19.6%). The whole basic characteristics of the participants are presented in Table 1.

**Table 1:** Demographic characteristics and basic information of the involved clinical and preventive medical students.

**Basic characteristics**	**Group A (*n* = 189) (%)**	**Group B (*n* = 46)(%)**	***P***
Gender			
Male	76 (40.2%)	10 (21.7%)	0.026^*^
Female	113 (59.8%)	36 (78.3%)	
Have systematically learned theoretical knowledge of PHE or not			
Yes	155 (82.0%)	44 (95.7%)	0.021^*^
No	34 (18.0%)	2 (4.3%)	
Have experienced rescue activity of PHE or not			
Yes	29 (15.3%)	0 (0%)	0.002^**^
No	160 (84.7%)	46 (100%)	
Have participated in emergency exercise of PHE or not			
Yes	61 (32.3%)	9 (19.6%)	0.107
No	128 (67.7%)	37 (80.4%)	

The chi-square test was used for comparing the basic characteristics of the groups. *P* < 0.05 is indicated in bold.

* *P* < 0.05,

** *P* < 0.01. Group A (n1 = 189) and group B (n2 = 46) are included. PHE, public health emergency.

### Comparison of the subjective attitudes toward PHE

**Figure 1:**
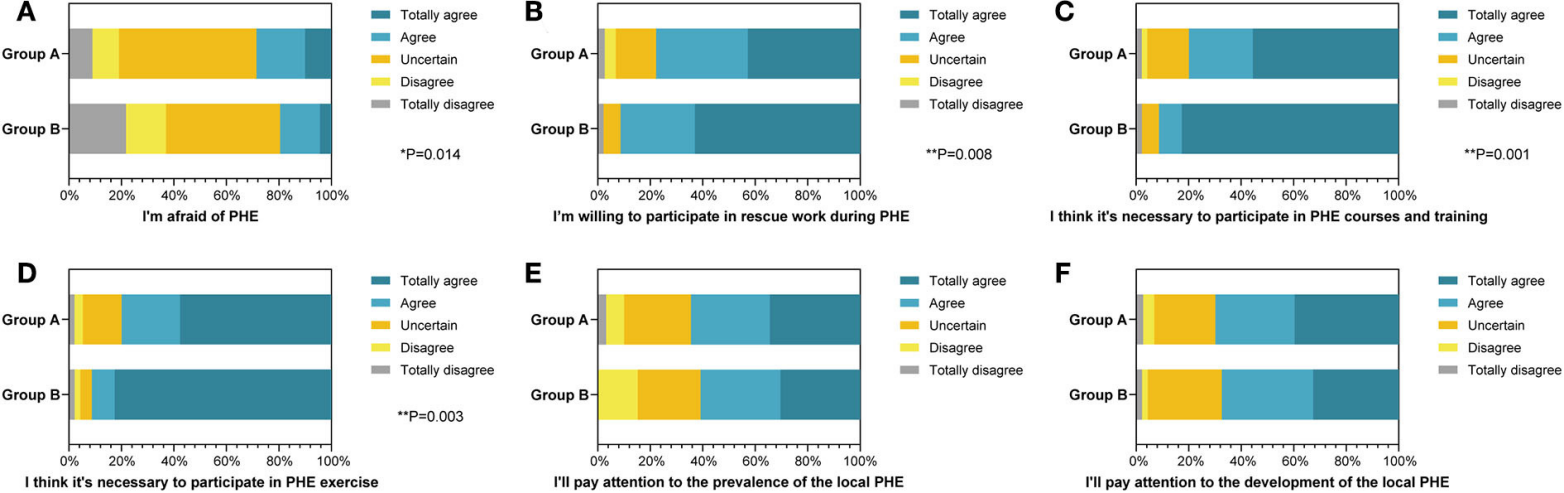
The percentage stacking histograms of the comparison in the subjective cognition toward public health emergency (PHE) between clinical and preventive medicine students with a 5-point Likert scale. **(A)** The fear of PHE between clinical and preventive medicine students. **(B)** The willingness to participate in rescue work during PHE between clinical and preventive medicine students. **(C)** The thought of the necessity to participate in PHE courses and training between clinical and preventive medicine students. **(D)** The thought of the necessity to participate in PHE exercise between clinical and preventive medicine students. **(E)** The attention to the prevalence of the local PHE between clinical and preventive medicine students. **(F)** The attention to the development of the local PHE between clinical and preventive medicine students. The nonparametric test was applied for the comparison (The number of group A is n1 = 189; the number of group B is n2 = 46).

To estimate the graduates' professional accomplishments, six queries were used to score them with a 5-point Likert scale. We prioritized their emotional responses toward PHE outbreaks, and the outcome revealed that clinical medicine graduates were less likely to be calm (19.0%) than preventive medicine graduates (37.0%, *P* = 0.014, Figure 1A). Gratifyingly, it was found that more than half of group A (77.8%) and most of group B (91.3%) were willing to participate in PHE rescue (*P* = 0.008, Figure 1B). Similarly, 79.9% of group A and 91.3% of group B thought PHE courses and training were necessary (*P* = 0.001, Figure 1C). Meanwhile, we found that several students in group B (91.3%) and more than half of group A students (79.9%) believed it was crucial to engage in PHE exercise (*P* = 0.003, Figure 1D). Overall, preventive medicine students showed more positive attitudes toward PHE rescue (*P* = 0.008), PHE courses and training (*P* = 0.001), and PHE exercise (P=0.003) than clinical medicine students (Figure 1). In both groups, most students considered they would pay attention to the prevalence (64.5 and 60.8%) and development (69.9 and 67.4%) of the local PHE, with no statistical significance detected. Table 2 summarizes the data for this section.

**Table 2:** Comparison of the subjective cognition in public health emergency between clinical and preventive medical students.

**Basic characteristics**	**Group A (*n* = 189) (%)**	**Group B (*n* = 46)(%)**	***P***
I'm afraid of PHE			
Totally disagree	17 (9.0%)	10 (21.7%)	0.014^*^
Disagree	19 (10.0%)	7 (15.3%)	
Uncertain	99 (52.4%)	20 (43.5%)	
Agree	35 (18.5%)	7 (15.2%)	
Totally agree	19 (10.1%)	2 (4.3%)	
I'm willing to participate in rescue work during PHE			
Totally disagree	5 (2.6%)	1 (2.2%)	0.008^**^
Disagree	8 (4.2%)	0 (0.0%)	
Uncertain	29 (15.3%)	3 (6.5%)	
Agree	66 (34.9%)	13 (28.3%)	
Totally agree	81 (42.9%)	29 (63.0%)	
I think it's necessary to participate in PHE courses and training			
Totally disagree	4 (2.1%)	1 (2.2%)	0.001^**^
Disagree	4 (2.1%)	0 (0.0%)	
Uncertain	30 (15.9%)	3 (6.5%)	
Agree	46 (24.3%)	4 (8.7%)	
Totally agree	105 (55.6%)	38 (82.6%)	
I think it's necessary to participate in PHE exercise			
Totally disagree	4 (2.1%)	1 (2.2%)	0.003^**^
Disagree	6 (3.2%)	1 (2.2%)	
Uncertain	28 (14.8%)	2 (4.3%)	
Agree	42 (22.2%)	4 (8.7%)	
Totally agree	109 (57.7%)	38 (82.6%)	
I'll pay attention to the prevalence of the local PHE			
Totally disagree	6 (3.2%)	0 (0.0%)	0.514
Disagree	13 (6.9%)	7 (15.3%)	
Uncertain	48 (25.4%)	11 (23.9%)	
Agree	57 (30.1%)	14 (30.4%)	
Totally agree	65 (34.4%)	14 (30.4%)	
I'll pay attention to the development of the local PHE			
Totally disagree	5 (2.6%)	1 (2.2%)	0.541
Disagree	8 (4.2%)	1 (2.2%)	
Uncertain	44 (23.3%)	13 (28.3%)	
Agree	57 (30.2%)	16 (34.8%)	
Totally agree	75 (39.7%)	15 (32.6%)	

The nonparametric test was used for the comparison. *P* < 0.05 is indicated in bold.

* *P* < 0.05,

** *P* < 0.01. Group A (n1 = 189) and group B (n2 = 46) are included. PHE, public health emergency.

### Comparison in the rescue knowledge and capabilities of PHE

According to the eighth edition of *Epidemiology* published by People's Health Publishing House, PHE is classified into four categories, namely, major communicable diseases, population unexplained diseases, major food and occupational poisoning, and other serious incidents affecting public health (2). For practical reasons, the “population unexplained diseases,” “major food and occupational poisoning,” and “other serious incidents affecting public health (natural disasters or serious traffic accidents)” were put into the “noncommunicable” category in this research.

**Figure 2:**
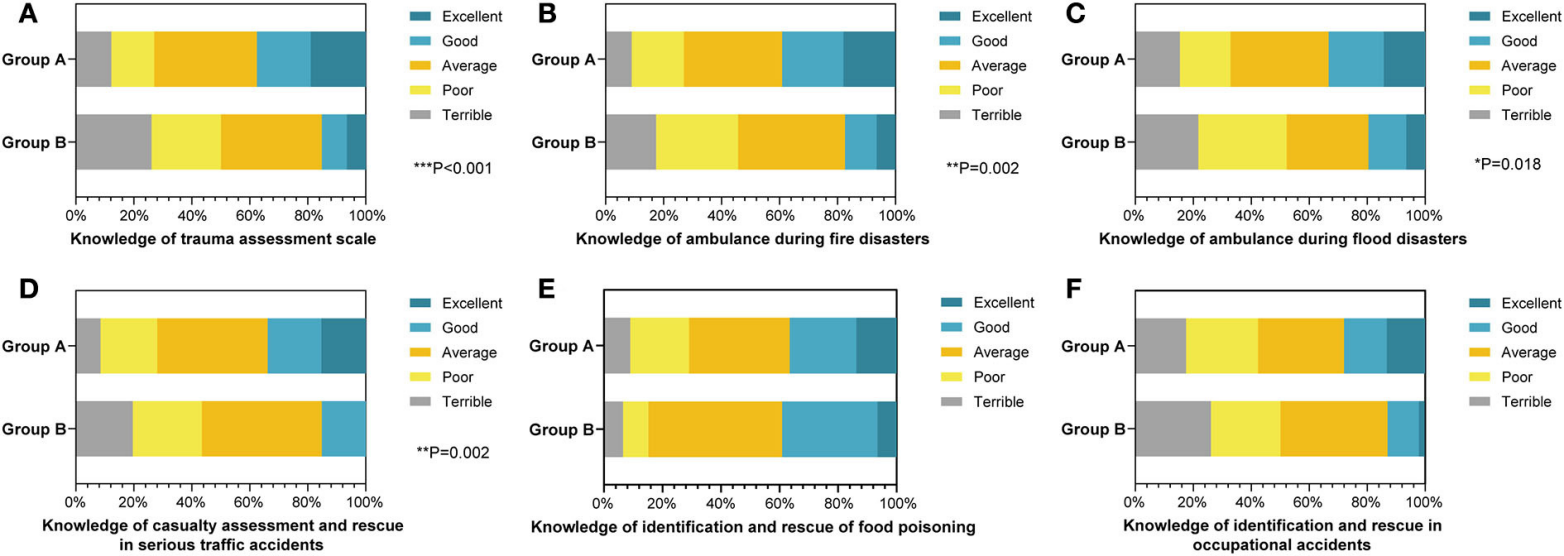
The percentage stacking histograms of the comparison in the knowledge of noncommunicable diseases associated with public health emergency (PHE) between clinical and preventive medicine students with a 5-point Likert scale. **(A)** The knowledge of trauma assessment scale between clinical and preventive medicine students. **(B)** The knowledge of ambulances during fire disasters between clinical and preventive medicine students. **(C)** The knowledge of ambulances during flood disasters between clinical and preventive medicine students. **(D)** The knowledge of casualty assessment and rescue in serious traffic accidents between clinical and preventive medicine students. **(E)** The knowledge of identification and rescue of food poisoning between clinical and preventive medical students. **(F)** The knowledge of identification and rescue in occupational accidents between clinical and preventive medical students. The nonparametric test was applied for the comparison (The number of group A is n1 = 189; the number of group B is n2 = 46).

To compare the two groups' theoretical knowledge and practical capabilities of noncommunicable diseases, the first-aid abilities were evaluated for quantification with a 5-point Likert scale (Table 3). Compared with group B (15.3%), group A had significantly better commands of the trauma assessment scale (37.5%, *P* < 0.001, Figure 2A). Consistently, in terms of the PHE caused by natural disasters such as fire disasters (*P* = 0.002, Figure 2B) and flood disasters (*P* = 0.018, Figure 2C), group A (39.2 and 33.3%) also afforded a firmer grasp of the ambulance skills than group B (17.4 and 19.5%). In terms of the knowledge of casualty assessment and rescue in serious traffic accidents, none of group B showed excellent competence, while 15% of group A declared that they excellently grasped therapies on serious traffic accident rescues (*P* = 0.002, Figure 2D). Nevertheless, the statistics indicated that the graduates showed an equivalent performance when assessing their knowledge of identification and rescue in food poisoning accidents (*P* = 0.436, Figure 2E). In addition, 53 in 189 students from group A considered they were good or excellent at identification and rescue of occupational accidents, but only 6 in 46 medicineshowed consistent attitude, although not statistically significant (*P* = 0.057, Figure 2F).

**Table 3:** Comparison of the knowledge of noncommunicable diseases associated with public health emergencies between clinical and preventive medical students.

**Basic characteristics**	**Group A (*n* = 189) (%)**	**Group B (*n* = 46)(%)**	***P***
Knowledge of trauma assessment scale			
Terrible	23 (12.2%)	12 (26.1%)	<0.001^***^
Poor	28 (14.8%)	11 (23.9%)	
Average	67 (35.5%)	16 (34.8%)	
Good	35 (18.5%)	4 (8.7%)	
Excellent	36 (19.0%)	3 (6.5%)	
Knowledge of ambulance during fire disasters			
Terrible	17 (9.0%)	8 (17.4%)	0.002^**^
Poor	34 (18.0%)	13 (28.3%)	
Average	64 (33.9%)	17 (37.0%)	
Good	40 (21.2%)	5 (10.9%)	
Excellent	34 (18.0%)	3 (6.5%)	
Knowledge of ambulance during flood disasters			
Terrible	29 (15.3%)	10 (21.7%)	0.018^*^
Poor	33 (17.5%)	14 (30.4%)	
Average	64 (33.9%)	13 (28.3%)	
Good	36 (19.0%)	6 (13.0%)	
Excellent	27 (14.3%)	3 (6.5%)	
Knowledge of casualty assessment and rescue in serious traffic accidents			
Terrible	16 (8.5%)	9 (19.6%)	0.002^**^
Poor	37 (19.6%)	11 (23.9%)	
Average	72 (38.1%)	19 (41.3%)	
Good	35 (18.5%)	7 (15.2%)	
Excellent	29 (15.3%)	0 (0%)	
Knowledge of identification and rescue of food poisoning			
Terrible	17 (9.0%)	3 (6.5%)	0.436
Poor	38 (20.1%)	4 (8.7%)	
Average	65 (34.4%)	21 (45.7%)	
Good	43 (22.7%)	15 (32.6%)	
Excellent	26 (13.8%)	3 (6.5%)	
Knowledge of identification and rescue in occupational accidents			
Terrible	33 (17.5%)	12 (26.1%)	0.057
Poor	47 (24.9%)	11 (23.9%)	
Average	56 (29.6%)	17 (37.0%)	
Good	28 (14.8%)	5 (10.9%)	
Excellent	25 (13.2%)	1 (2.1%)	

The nonparametric test was applied for the comparison. *P* < 0.05 is indicated in bold.

* *P* < 0.05,

** *P* < 0.01,

*** *P* < 0.001. Group A (n1 = 189) and group B (n2 = 46) are included.

**Figure 3:**
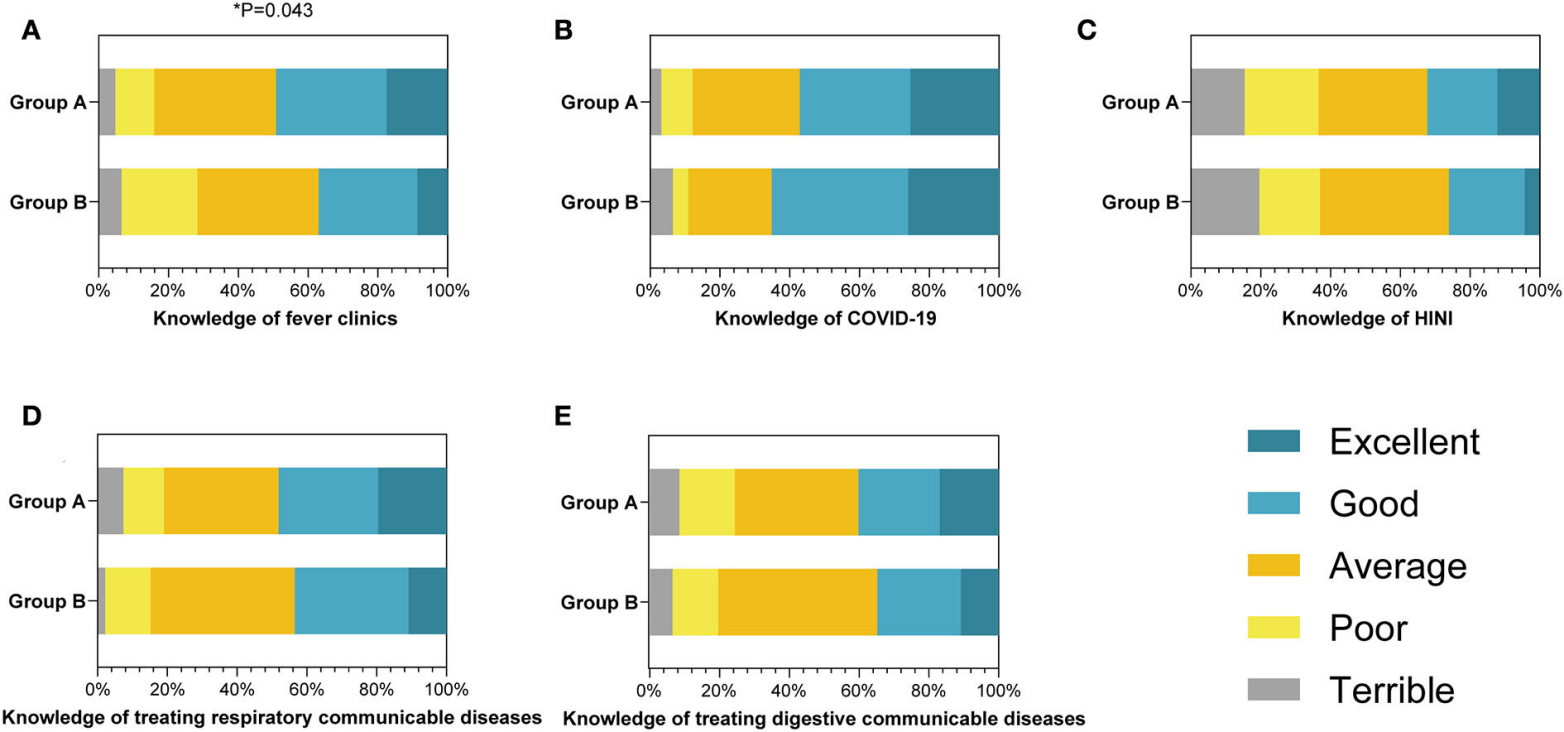
The percentage stacking histograms of the comparison in the knowledge of communicable diseases associated with public health emergency (PHE) between clinical and preventive medicine students with a 5-point Likert scale. **(A)** The knowledge of fever clinics between clinical and preventive medicine students. **(B)** The knowledge of COVID-19 between clinical and preventive medicine students. **(C)** The knowledge of H1N1 between clinical and preventive medicine students. **(D)** The knowledge of treating respiratory communicable diseases between clinical and preventive medicine students. **(E)** The knowledge of treating digestive communicable diseases between clinical and preventive medicine students. The nonparametric test was applied for the comparison (The number of group A is n1 = 189; the number of group B is n2 = 46).

Concerning communicable diseases, derived epidemiological questions were employed to appraise their response capabilities (Table 4). The results pointed out that there was no difference between the groups' capabilities in general. Nearly half (49.2%) of group A and 17 of 46 from group B indicated good or excellent prehension of fever clinics with statistical significance (*P* = 0.043, Figure 3A). Nonetheless, both groups mastered sufficient competency to respond to major communicable diseases such as COVID-19 (Figure 3B) or H1N1 (Figure 3C). In detail, more than half of the students from group A (57.1%) and group B (65.2%) displayed that they had adequate competence to respond to COVID-19. Concurrently, 61 out of 189 students from group A and 12 out of 46 students from group B considered they had mastered sufficient skills to cope with H1N1. They also showed equal apprehensions in responding to respiratory communicable diseases (Figure 3D) and digestive communicable diseases (Figure 3E).

**Table 4:** Comparison of the knowledge of communicable diseases associated with public health emergencies between clinical and preventive medical students.

**Basic characteristics**	**Group A (*n* = 189) (%)**	**Group B (*n* = 46)(%)**	***P***
Knowledge of fever clinics			
Terrible	9 (4.8%)	3 (6.5%)	0.043^*^
Poor	21 (11.1%)	10 (21.7%)	
Average	66 (34.9%)	16 (34.8%)	
Good	60 (31.7%)	13 (28.3%)	
Excellent	33 (17.5%)	4 (8.7%)	
Knowledge of COVID-19			
Terrible	6 (3.2%)	3 (6.5%)	0.544
Poor	17 (9.0%)	2 (4.4%)	
Average	58 (30.7%)	11 (23.9%)	
Good	60 (31.7%)	18 (39.1%)	
Excellent	48 (25.4%)	12 (26.1%)	
Knowledge of HINI			
Terrible	29 (15.3%)	9 (19.6%)	0.418
Poor	40 (21.2%)	8 (17.4%)	
Average	59 (31.2%)	17 (37.0%)	
Good	38 (20.1%)	10 (21.7%)	
Excellent	23 (12.2%)	2 (4.3%)	
Knowledge of treating respiratory communicable diseases			
Terrible	14 (7.4%)	1 (2.2%)	0.634
Poor	22 (11.6%)	6 (13.0%)	
Average	62 (32.8%)	19 (41.3%)	
Good	54 (28.6%)	15 (32.6%)	
Excellent	37 (19.6%)	5 (10.9%)	
Knowledge of treating digestive communicable diseases			
Terrible	16 (8.5%)	3 (6.5%)	0.768
Poor	30 (15.9%)	6 (13.0%)	
Average	67 (35.4%)	21 (45.7%)	
Good	44 (23.3%)	11 (23.9%)	
Excellent	32 (16.9%)	5 (10.9%)	

The nonparametric test was applied for the comparison. *P* < 0.05 is indicated in bold.

* *P* < 0.05. Group A (n1 = 189) and group B (n2 = 46) are included.

Together, the consequence displayed that, although clinical medical students performed better in noncommunicable diseases, preventive medicine students presented equal capabilities in confronting communicable diseases.

### Comparison in the mastery of PHE regulations and psychological intervention abilities

**Figure 4:**
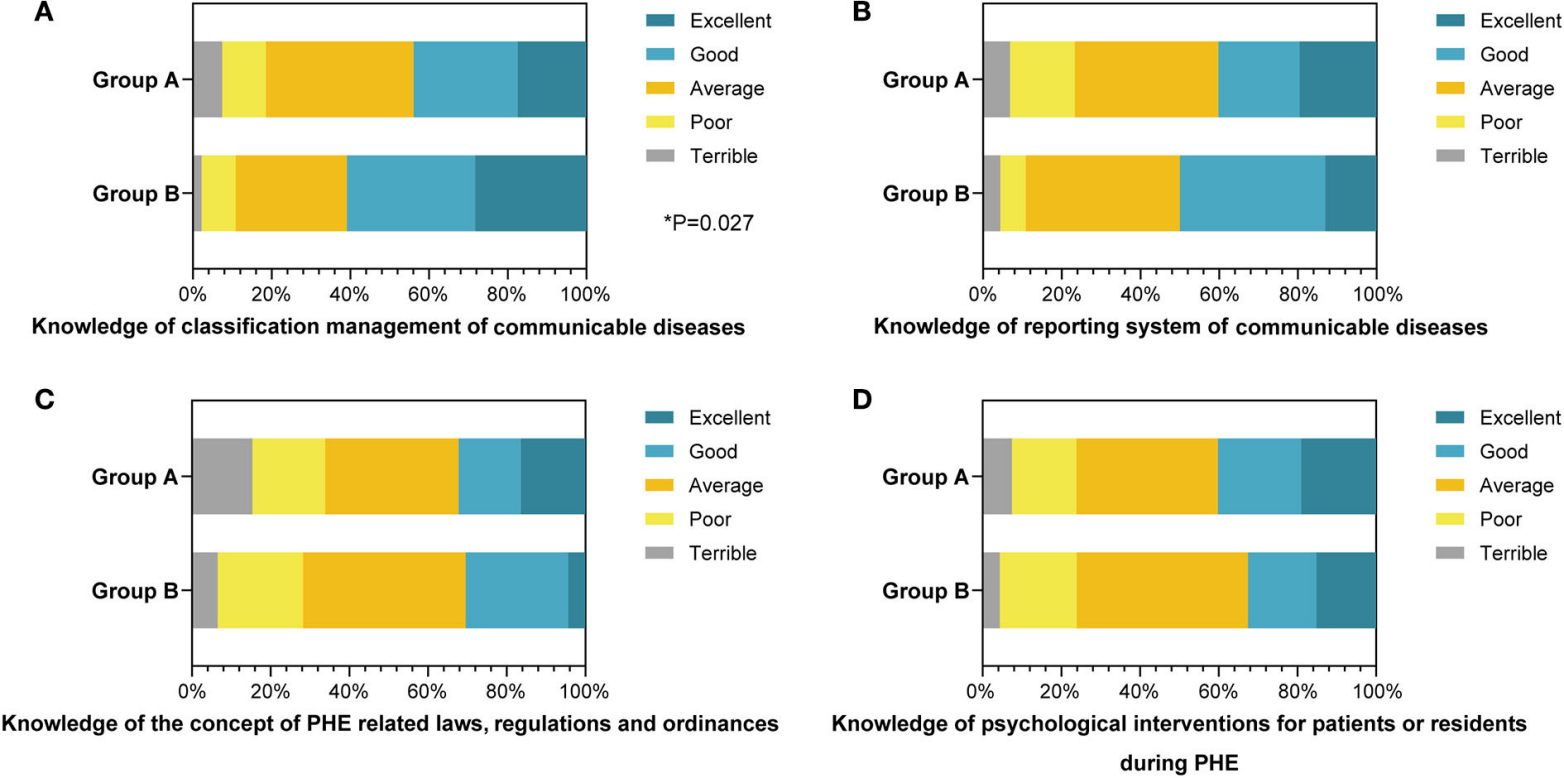
The percentage stacking histograms of the comparison in the mastery of public health emergency (PHE) regulations and psychological intervention abilities between clinical and preventive medical students with a 5-point Likert scale. **(A)** The knowledge of classification management of communicable diseases between clinical and preventive medical students. **(B)** The knowledge of reporting system of communicable diseases between clinical and preventive medical students. **(C)** The knowledge of the concept of PHE-related laws, regulations, and ordinances between clinical and preventive medical students. **(D)** The knowledge of psychological interventions for patients or residents during PHE between clinical and preventive medicine students. The nonparametric test was applied for the comparison (The number of group A is n1 = 189; the number of group B is n2 = 46).

During the response of PHE, the medical workers should register and report the cases except for conducting emergency rescues. Thus, it is significant for them to command sufficient knowledge related to PHE besides rescue knowledge. We further investigated their mastery of the PHE regulations and psychological intervention abilities (Table 5). Group B students were more familiar with the PHE classification management (60.8%) than group A students (44.0%), fully in line with our expectation (*P* = 0.027, Figure 4A). Conjointly, 50% of students in group B showed positive (good or excellent) attitudes toward their cognition of the PHE reporting system, which exceeded the corresponding percentage of group A (40.2%, Figure 4B), although not statistically different. No significant differences were found between the two groups for their commands of PHE-related laws, regulations, and ordinances (Figure 4C). When they intervened in the psychological states of the patients, 23.8% of group A and 23.9% of group B declared a negative (terrible or poor) performance (*P* = 0.570, Figure 4D).

**Table 5:** Comparison of the mastery of public health emergency regulations and psychological intervention abilities between clinical and preventive medical students.

**Basic characteristics**	**Group A (*n* = 189) (%)**	**Group B (*n* = 46)(%)**	***P***
Knowledge of classification management of communicable diseases			
Terrible	14 (7.4%)	1 (2.2%)	0.027^*^
Poor	21 (11.1%)	4 (8.7%)	
Average	71 (37.5%)	13 (28.3%)	
Good	50 (26.5%)	15 (32.5%)	
Excellent	33 (17.5%)	13 (28.3%)	
Knowledge of reporting system of communicable diseases			
Terrible	13 (6.9%)	2 (4.4%)	0.269
Poor	31 (16.4%)	3 (6.5%)	
Average	69 (36.5%)	18 (39.1%)	
Good	39 (20.6%)	17 (37.0%)	
Excellent	37 (19.6%)	6 (13.0%)	
Knowledge of the concept of PHE related laws, regulations, and ordinances			
Terrible	29 (15.3%)	3 (6.5%)	0.904
Poor	35 (18.5%)	10 (21.7%)	
Average	64 (33.9%)	19 (41.3%)	
Good	30 (15.9%)	12 (26.1%)	
Excellent	31 (16.4%)	2 (4.4%)	
Knowledge of psychological interventions for patients or residents during PHE			
Terrible	14 (7.4%)	2 (4.3%)	0.570
Poor	31 (16.4%)	9 (19.6%)	
Average	68 (36.0%)	20 (43.5%)	
Good	40 (21.2%)	8 (17.4%)	
Excellent	36 (19.0%)	7 (15.2%)	

The nonparametric test was applied for the comparison. *P* < 0.05 is indicated in bold.

* *P* < 0.05. Group A (n1 = 189) and group B (n2 = 46) are included. PHE, public health emergency.

## Discussion

Public health emergency has received considerable attention in recent years due to the globally unprecedented outbreaks of COVID-19, a communicable public health disease (3, 17, 18). It is a Chinese characteristic and advantage to convene clinical departments and public health centers to establish the Joint Prevention and Control Mechanism for the response of the PHE (19–21). However, during the early stages of the Chinese fight against the epidemic, the discrepancies in PHE expertise and insufficient public health knowledge of clinicians caused many adverse effects on the Chinese PHE coping system (22). In this research on the clinical and preventive medical graduates' attitudes toward PHE and relevant comprehensive knowledge, we found that the discrepancies in the Chinese PHE system mentioned above were likely caused by the separation of clinical and preventive education systems as well as the scarcity of PHE practical training in the Chinese medical education systems. The results showed that the clinical medicine graduates were not concerned enough about the PHE courses and training, but they well mastered the knowledge of PHE rescue works. Meanwhile, the preventive graduates were more willing to participate in PHE rescues, but they lacked practical rescue knowledge and practical capabilities compared with clinical students. In addition, more PHE training and exercise might help the graduates master PHE knowledge and practical capabilities preferably. As reported, the eruption of nosocomial infections in the early stage of the COVID-19 epidemic revealed the lack of public health knowledge of clinicians; meanwhile, frontline doctors had to bear overload with the increasing number of infected patients due to the scarcity of frontline health workers in the early stage of COVID-19 epidemic (23–25). To strengthen medical students' PHE knowledge and practical capabilities and change the situation of medical and preventive separation from the educational stage, which was expected to further optimize the Chinese PHE emergency management system, we recommend the Chinese medical education systems implement the integration of preventive and clinical medicine education and increment public health practice training and exercise as well (11, 26).

### Clinical medical education reform: Adding preventive medicine practice

It was gratifying that, in terms of the subjective cognition of the PHE, most students in both groups stated that they would pay continuous attention to the prevalence and development of the local PHE, revealing the great professional responsibilities stimulated by the existing Chinese medical education system. However, opinions seemed to differ when talking about the concerns of PHE courses, training, exercise, and rescue. The preventive students tended to attach more significance to the PHE training than clinical medicine students. A possible explanation for this might be that with the consensual impression of a relatively weaker requirement of clinical professional knowledge during ordinary public health rescue, the clinical students and clinicians prefer to distribute more vigor upon the core clinical affairs such as emergency, clinic, and section, instead of ordinary public health events (15, 27). Similarly, Wiwanitkit et al. also discovered that preventive medicine and healthcare professionals were considered to act as the main force toward normal public emergencies, which probably induced preventive medicine students to emphasize PHE training regarding as their professional duty (28, 29). Nonetheless, the emerging epidemic of COVID-19 exposed the detrimental effects of insufficient attention to public health knowledge among clinicians. Minutely, failure to identify COVID-19's infectivity promptly and inadequate self-protection awareness among medical staff were the main manifestations (22, 24, 25). Under this circumstance, the clinical training program is expected to include methods like 1) incorporating emergency training and exercise into scoring courses and 2) increasing the amount and categories of preventive training and exercise in the undergraduate curriculum system (30). This would undoubtedly help the clinical students vividly recognize the importance of public health knowledge. Therefore, they will be able to maintain public health when they deal with PHE as clinicians.

### Preventive medical education reform: Strengthening first-line rescue capability

As stated previously, the PHEs were classified into two categories in this study. The statistic showed that clinical graduates had a better command of noncommunicable disease rescue skills, including treatment for trauma caused by natural disasters or serious traffic accidents. As for the reason for this phenomenon, we speculated the fact that fewer credits were attached to the courses like surgery for the preventive students in their undergraduate training program. Taking the Xiangya Medical School as an example, the surgery, in which the students were expected to learn the therapies for noncommunicable diseases rescue, occupies 7 credits in the clinical medicine curriculum but only 2 credits for the preventive medicine students (Supplementary Tables 1, 2). Indicating that the training program for the preventive medicine students paid less attention to basic clinical courses than preventive specialized courses (31). In light of this training program, the preventive medicine graduates cannot put themselves forward when the number of outbreak cases surged and clinicians were seriously insufficient, even if they had exactly learned the same basic courses. Consequently, the preventive medicine training program is expected not only to pay attention to cultivating specific preventive professional skills but also to establish an integrated medical education system as the concept of “global minimum essential requirements (GMER)” developed by the Institute for International Medical Education (IIME), for graduates of preventive medicine to participate in first-line rescues when PHE occurs (20, 32). Concerning the knowledge of communicable diseases such as COVID-19 or H1N1, we were surprised to find that both groups had mastered sufficient competency whether from clinical medicine or preventive medicine. After comprehensively analyzing their mastery of the noncommunicable and communicable diseases, we derived the conjecture that in the epidemic environment every medical professional emphasized their ability to cope with communicable diseases. As Liu et al. discovered although deficient in noncommunicable diseases, the preventive medicine graduates presented equal capabilities in confronting communicable diseases with clinical graduates (33). Thus, it is feasible to keep promoting clinical rescue skills of preventive medical students during the undergraduate stage, expecting to cultivate preventive experts who have the competence to join the clinicians disposing of the first-line PHE rescue in future emergencies.

### Medical education reform: Cultivating cross-disciplinary medical talents

As the review research by Zhu et al. reported, the new medical background calls for compound medical talents and medical education reform that systematically integrates medical knowledge, including clinical diagnosis and treatment, population health services, medical regulations, as well as psychological intervention abilities (34). In terms of the medical education reform under the background of PHE, we further compared their mastery of PHE regulations and their abilities to intervene in the patients' psychologies during PHE between the clinical and preventive graduates. In addition to the attitudes, knowledge, and capabilities of the PHE, these supplements might reflect their whole cognition of the PHE system. The results showed that the preventive graduates were more familiar with the PHE classification and reporting systems, but they shared equal performance when the PHE laws, regulations, ordinances, and psychological intervention abilities were mentioned. The explanation as to why some of the graduates ultimately failed to grasp enough knowledge of the PHE reporting systems and psychological intervention abilities was the specific course arrangements of their training programs. For example, the corresponding knowledge of PHE regulations and psychological counseling was taught in Health Law and Medicopsychology, some of which served as compulsory courses but others were elective courses for the undergraduates (15). Accordingly, there would remain a total knowledge blind zone for the graduates who did not take any kind of this elective course, while others would only grasp fragmentary knowledge despite taking a couple of specific courses. It is recommended to increase the number of compulsory courses that teach PHE regulations and psychological interventions in the medical training program, allowing them to form a complete impression. Equipping them with basic correlative knowledge would benefit them when dealing with PHE problems (35).

### Comparison and features

There were a few pieces of literature that also studied the medical students' rescue knowledge and capabilities in PHE. The researchers reached the same conclusion that most clinical medicine students lacked the knowledge of PHE, emphasizing the importance of cultivating the clinical medicine students' knowledge and capabilities of PHE, which was consistent with our study (36). They also considered that preventive medicine students commanded better PHE knowledge than clinical medicine students (37). In this research, however, we discovered that clinical medicine students performed better in noncommunicable diseases than preventive medicine students, suggesting the focus of training programs should not only concentrate on communicable diseases. Moreover, this study investigated the medical students' subjective cognition toward PHE and their cognition of PHE laws and regulations, which generated a more comprehensive suggestion.

To sum up, we believe that the current clinical and preventive medicine education should be improved in the following directions: (1) To further implement the integration of preventive and clinical medical education by minutely strengthening the basic knowledge and capabilities of clinical PHE rescue among preventive medicine students while upgrading the status of preventive medicine courses in the clinical training programs to help them identify the importance of public health knowledge. (2) To increase the amounts and categories of emergency training and exercises and incorporate emergency training and exercises into scoring courses in both clinical and preventive training programs. (3) To cultivate compound medical talents mastering, not only medical knowledge but also psychological and legal knowledge, the medical education system should pay equal attention to the medical students' comprehensive qualities as well as their professional capabilities. Frankly, our research also has some limitations that need enhancement in the following aspects: (1) the sample size is insufficient with only one school to investigate. Multicenter research can be supplemented in the future and (2) this research is a cross-sectional study, and a cohort or retrospective study may be considered in the future.

## Conclusion

This was the first study to explore the direct impact of the training programs on the clinical and preventive medical students in the subjective cognition in PHE, relevant knowledge, and coping abilities. We found discrepancies in these aspects between the two groups, showing the majority of medical students have barely systematically mastered PHE-related knowledge, which helped better devise the implementation during the medical education reform. These data also demonstrated that clinical medical students mastered a higher level of coping abilities for noncommunicable diseases. Meanwhile, preventive medical students were more familiar with the PHE classification and reporting system and showed a greater willingness in rescuing during PHE. However, clinical and preventive medical students' response abilities to communicable diseases showed no significant difference with the same attention to the development of the local PHE. Generally speaking, to consolidate the weak points in the Chinese PHE emergency management system and meet the best educational and medical demands sufficiently, equal attention should be paid to the medical students with different majors along with a complete integrated PHE curriculum.

## Data availability statement

The original contributions presented in the study are included in the article/Supplementary material, further inquiries can be directed to the corresponding authors.

## Author contributions

JW and PR: questionnaire design and questionnaire survey. YY and YQ: data sorting, statistical analysis, and manuscript writing. YL: manuscript revision and paper conception. ZY and PW: manuscript revision. All authors contributed to the article and approved the submitted version.

## Funding

This study was supported by the 2021 Innovation and Entrepreneurship Educational Reform Project of Central South University (Nos. 61 and 62) and the Educational Reform Project of Central South University (2020jy179).

## Conflict of interest

The authors declare that the research was conducted in the absence of any commercial or financial relationships that could be construed as a potential conflict of interest.

## Publisher's note

All claims expressed in this article are solely those of the authors and do not necessarily represent those of their affiliated organizations, or those of the publisher, the editors and the reviewers. Any product that may be evaluated in this article, or claim that may be made by its manufacturer, is not guaranteed or endorsed by the publisher.
