# Supplementary material for: A cross-sectional study on the response abilities of clinical and preventive medical students in public health emergency
Source: Front Public Health. 2022 Dec 1;10:1017063. doi: 10.3389/fpubh.2022.1017063 (PMC9751026; doi:10.3389/fpubh.2022.1017063)
Supplement: Supplementary file 1 [file Table_1.DOCX]

Supplementary Material

# Supplementary Tables

**Supplementary TABLE 1** Training program for clinical medical students.

| **Categories of curriculums** | **Names of courses** | **Properties of courses** | **Credits** |
| --- | --- | --- | --- |
| **Subject fundamental curriculum** | Neurobiology | Elective | 1.5 |
|  | Medical Development Biology | Elective | 1 |
|  | Medical Parasitology | Compulsory | 2 |
|  | Biochemistry C | Compulsory | 3.5 |
|  | Biochemistry Experiment C | Compulsory | 1.5 |
|  | Introduction to Biotechnology | Elective | 1 |
|  | Molecular Biology C | Compulsory | 1.5 |
|  | Molecular Biology Experiment C | Elective | 0.5 |
|  | Medical Genetics | Compulsory | 1.5 |
|  | Advances on Medical Genetics | Elective | 1 |
|  | Medical Ethics | Compulsory | 2 |
|  |  |  |  |
| **Professional core curriculum** | Clinical Practice A | Compulsory | 46 |
|  | Community Health Service | Compulsory | 2 |
|  | Diagnostics A | Compulsory | 5 |
|  | Medical Imaging A | Compulsory | 2.5 |
|  | Operative Surgery | Compulsory | 1.5 |
|  | Internal Medicine A | Compulsory | 8.5 |
|  | General Surgery | Compulsory | 2 |
|  | Surgery A | Compulsory | 7 |
|  | Obstetrics and Gynecology A | Compulsory | 4 |
|  | Pediatrics A | Compulsory | 4 |
|  | Infectious Diseases A | Compulsory | 2.5 |
|  | Ophthalmology A | Compulsory | 1.5 |
|  | Otorhinolaryngology, Head and Neck Surgery A | Compulsory | 1.5 |
|  | Dermatovenereology A | Compulsory | 2 |
|  | Neurology A | Compulsory | 2 |
|  | Stomatology A | Compulsory | 1.5 |
|  | Psychiatry A | Compulsory | 2 |
|  | Regional Anatomy | Compulsory | 3.5 |
|  | Systematic Anatomy A | Compulsory | 3.5 |
|  | Histology and Embryology A | Compulsory | 3 |
|  | Physiology A | Compulsory | 5 |
|  | Pathophysiology A | Compulsory | 3 |
|  | Medical Immunology B | Compulsory | 2.5 |
|  | Functional Experiment A3-Pharmacology Experiment | Compulsory | 1 |
|  | Functional Experiment A2-Pathophysiology Experiment | Compulsory | 1 |
|  | Functional Experiment A1-Physiology Experiment | Compulsory | 1.5 |
|  | Pharmacology C | Compulsory | 3.5 |
|  |  |  |  |
| **Professional curriculum** | Introduction to Clinical Medicine | Compulsory | 1 |
|  | Early Clinical Contact | Compulsory | 1 |
|  | Chinese Medicine | Compulsory | 1.5 |
|  | Pathology A | Compulsory | 5 |
|  | Medical Microbiology B | Compulsory | 3 |
|  | Introduction to General Practice | Compulsory | 1 |
|  | Medical Statistics B | Compulsory | 2 |
|  | Epidemiology B | Compulsory | 2 |
|  | Evidence-based Medicine | Compulsory | 1 |
|  | Hygiene A | Compulsory | 2.5 |
|  |  |  |  |
| **Professional elective curriculum** | Science of Communication Skills | Elective | 1 |
|  | Laboratory Animal Science | Elective | 2 |
|  | Emergency Medicine | Elective | 1 |
|  | Nuclear Medicine | Elective | 1 |
|  | Rehabilitation Medicine | Elective | 1 |
|  | Medical Psychology | Elective | 1 |
|  | Behavioral Medicine | Elective | 1 |
|  | Geriatric Medicine | Elective | 1 |
|  | Pain Medicine | Elective | 1 |
|  | Forensic Medicine | Elective | 1 |
|  | Ultrastructure and Ultrastructural Pathology in Medicine B | Elective | 1.5 |
|  | Study and Design of Medical Science | Elective | 1 |
|  | Science of Health Education | Elective | 1 |
|  | Drug Toxicology | Elective | 1 |
|  | Health Law B | Elective | 1 |
|  | Health Care Administration | Elective | 1 |
|  | Health Economics | Elective | 1 |
|  | Social Medicine B | Elective | 1 |
|  | Subject Lecture on Modern Molecular Biology | Elective | 1 |

**Supplementary TABLE 2** Training program for preventive medical students.

| **Categories of curriculums** | **Names of courses** | **Properties of courses** | **Credits** |
| --- | --- | --- | --- |
| **Subject fundamental curriculum** | Diagnostics B | Compulsory | 4 |
|  | Science of Communication Skills | Compulsory | 1 |
|  | Medical Imaging B | Compulsory | 1.5 |
|  | Internal Medicine B | Compulsory | 4 |
|  | Obstetrics and Gynecology B | Compulsory | 1.5 |
|  | Pediatrics B | Compulsory | 1.5 |
|  | Infectious Diseases A | Compulsory | 2.5 |
|  | Ophthalmology | Elective | 1 |
|  | Otorhinolaryngology, Head and Neck Surgery B | Elective | 1 |
|  | Dermatovenereology A | Compulsory | 2 |
|  | Neurology B | Compulsory | 1 |
|  |  |  |  |
| **Professional core curriculum** | Medical Psychology | Elective | 1 |
|  | Behavioral Medicine | Elective | 1 |
|  | Geriatric Medicine | Elective | 1 |
|  | Stomatology B | Elective | 1 |
|  | Psychosis and Orthopsychiatry | Compulsory | 1 |
|  | Systematic Anatomy A | Compulsory | 3.5 |
|  | Histology and Embryology B | Compulsory | 2.5 |
|  | Physiology B | Compulsory | 3.5 |
|  | Pathology B | Compulsory | 3.5 |
|  | Pathophysiology B | Compulsory | 2 |
|  | Medical Immunology A | Compulsory | 3.5 |
|  | Medical Microbiology C | Compulsory | 2 |
|  | Medical Parasitology | Compulsory | 2 |
|  | Functional Experiment B2-Pharmacology Experiment | Compulsory | 1 |
|  | Pharmacology E | Compulsory | 3 |
|  | Cell Biology C | Compulsory | 2 |
|  | Cell Biology Experiment C | Compulsory | 1 |
|  | Process in Biology | Elective | 1 |
|  | Biochemistry C | Compulsory | 3.5 |
|  | Biochemistry Experiment C | Compulsory | 1.5 |
|  | Introduction to Biotechnology | Elective | 1 |
|  | Molecular Biology C | Compulsory | 1.5 |
|  | Subject Lecture on Modern Molecular Biology | Elective | 1 |
|  | Medical Genetics | Elective | 1.5 |
|  | Medical Ethics | Compulsory | 2 |
|  | Information Retrieval C | Elective | 1 |
|  | General Surgery | Compulsory | 2 |
|  |  |  |  |
| **Professional curriculum** | Neurobiology | Elective | 1.5 |
|  | Functional Experiment B1-Physiology and Pathophysiology Experiment | Compulsory | 1 |
|  | Epidemiology A | Compulsory | 4.5 |
|  | Health Statistics | Compulsory | 4.5 |
|  | Occupational Health and Occupational Medicine | Compulsory | 4.5 |
|  | Environmental Hygiene | Compulsory | 4 |
|  | Nutrition and Food Hygiene | Compulsory | 4 |
|  | Child and Adolescent Health | Compulsory | 3 |
|  | Maternal and Child Health | Elective | 1.5 |
|  | Toxicology | Compulsory | 3 |
|  |  |  |  |
| **Professional elective curriculum** | Social Medicine | Compulsory | 2 |
|  | Health Care Administration | Compulsory | 1 |
|  | Health Law A | Compulsory | 1.5 |
|  | Sanitary Chemistry | Compulsory | 4 |
|  | Chinese Medicine | Elective | 1.5 |
|  | An Introduction to Public Health and Preventive Medicine | Compulsory | 1 |
|  | Study and Design of Medical Science | Compulsory | 1 |
|  | Health Supervision | Elective | 1 |
|  | Public Health Front Lecture | Elective | 1 |
|  | Medical Statistical Software and Application | Elective | 1 |
|  | Evidence-based Mediciney | Elective | 1 |
|  | Radio-hygiology | Elective | 1 |
|  | Modern Nutriology | Elective | 1 |
|  | Science of Health Education | Elective | 1 |
|  | Toxicological Evaluation of Health-related Products | Elective | 1 |
|  | Health Economics | Elective | 1 |
|  | Orthopsychiatry | Elective | 1 |
|  | Foundation of Sanitary Inspection | Elective | 1 |

**
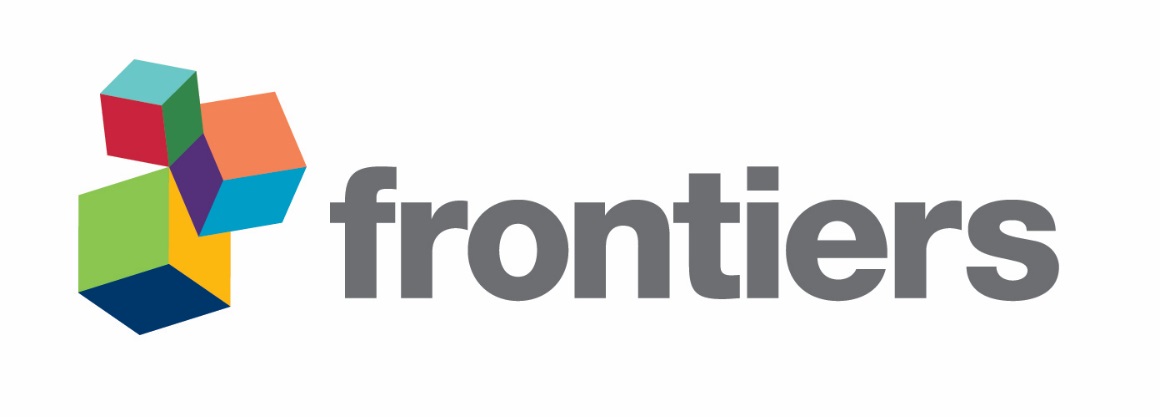
**
